# How does contextual information affect aesthetic appreciation and gaze behavior in figurative and abstract artwork?

**DOI:** 10.1167/jov.24.12.8

**Published:** 2024-11-08

**Authors:** Soazig Casteau, Daniel T. Smith

**Affiliations:** 1 Department of Psychology, Durham University, Durham, UK

**Keywords:** aesthetic experience, gaze behavior, contextual information, figurative and abstract artwork

## Abstract

Numerous studies have investigated how providing contextual information with artwork influences gaze behavior, yet the evidence that contextually triggered changes in oculomotor behavior when exploring artworks may be linked to changes in aesthetic experience remains mixed. The aim of this study was to investigate how three levels of contextual information influenced people's aesthetic appreciation and visual exploration of both abstract and figurative art. Participants were presented with an artwork and one of three contextual information levels: a title, title plus information on the aesthetic design of the piece, or title plus information about the semantic meaning of the piece. We measured participants liking, interest and understanding of artworks and recorded exploration duration, fixation count and fixation duration on regions of interest for each piece. Contextual information produced greater aesthetic appreciation and more visual exploration in abstract artworks. In contrast, figurative artworks were highly dependent on liking preferences and less affected by contextual information. Our results suggest that the effect of contextual information on aesthetic ratings arises from an elaboration effect, such that the viewer aesthetic experience is enhanced by additional information, but only when the meaning of an artwork is not obvious.

## Introduction

Neuroaesthetics (Chatterjee & Vartanian, 2014; Zeki, 1999) combines neuroscience and the study of aesthetics, with the goal of understanding the neural mechanisms underlying aesthetic experiences, including the perception and appreciation of art, music, and other forms of aesthetic stimuli. Bullot and Reber (2013) draw on a psychohistorical perspective suggesting that aesthetic appreciation occurs at one of three levels, depending on the perceiver's contextual awareness. The first level is referred to as *basic exposure*, which happens when aesthetic appreciation (i.e., the degree to which a stimulus is appreciated because of its beauty, or some other factor associated with aesthetic preference) is based purely on the visual properties of the artwork. Bullot and Reber (2013) acknowledge that in the absence of contextual knowledge (either prior knowledge or information provided alongside the artwork) viewers may rely on a process of *mindreading,* where the viewer attempts to understand the thoughts and intentions of the artist (Carruthers, 2009; Nichols & Stich, 2003) to generate a context that allows them to interpret the work. However, they also argue this information may not always be entirely accurate, potentially leading to difficulties interpreting the visual elements and impeding appreciation. The second level of the model is the *artistic design stance*, which refers to the development of knowledge around the artwork and happens when the perceiver is given precise contextual information, such as the making, authorship, and functions of artworks. Once the artistic design stance is fully adopted the contextual information allows viewers to evoke deeper emotional responses to the piece the third level is reached, what Bullot and Reber refers to as the “artistic understanding.” This final level involves the comprehension of the meaning, function, or artistic status of an artwork.

The three-stage model draws on several lines of evidence. First, studies have shown that basic aesthetic judgements can be formed at an early, largely sensory stage of appreciation (e.g., Bachmann & Vipper, 1983). Verhavert, Wagemans, and Augustin (2018) looked at the time course of aesthetic experience. They presented reproductions of paintings to their participants varying the presentation times (from 10 to 500 ms) and asked them to rate the paintings using three different scale (beauty, specialness, and impressiveness). Their results suggest that basic aesthetic judgments (i.e., beauty and specialness) can be formed as fast as 30 ms. Second, it has been shown that individuals tend to appreciate artworks more when they are aware of the effort that was invested in creating them, which Kruger, Wirtz, Van Boven, and Altermatt (2004) refer to as the *effort heuristic*, and that experimentally manipulating the availability of contextual information affects aesthetic judgements. For example, Gerger and Leder (2015) presented abstract, semiabstract, and figurative artworks and manipulated titles so that they were either absent (no title), semantically matching, or semantically non-matching. They observed that participants gave lower in ratings for non-matching titles compared to matching and absent titles. Similarly, Bailey-Ross, Beresford, Smith, and Warwick (2019) showed that participants reported greater liking for figurative paintings presented with a meaningful context that for those presented with a title but no context. In a recent study, Williams, McSorley, and McCloy (2020) reported that the artist's gesture (e.g., stippling, stroking) influenced an individual's engagement with the artworks, suggesting that the understanding of the creative process influences aesthetic appreciation. Swami (2013) investigated whether the model of Bullot and Reber (2013) could account for aesthetic appreciation across different genres of art by manipulating the information accompanying the paintings of Max Ernst (i.e., no information, title and author and broad contextual information, and content-specific contextual information). Contextual information did indeed increase aesthetic understanding and appreciation, and higher ratings were observed in the content-specific information condition. In a second experiment Swami (2013) observed that when there was no contextualizing information, participants’ aesthetic appreciation was higher for figurative compared to abstract art (see also Moore & West, 2012). However, in contrast to the first experiment, contextual information resulted in increased understanding and liking/ interest of abstract but not figurative artworks. One likely explanation for the failure to observe an effect of contextual information on liking in figurative painting is that naïve observers tend to prefer figurative art and find it easier to understand (Moore & West, 2012; Szubielska, Francuz, Niestorowicz, & Bałaj, 2018), thus reducing the relevance of the contextual information. Consistent with this explanation, Millis (2001) reported that contextual information only enhances a viewer's appreciation when it helps the viewer to contextualize and understand the painting beyond its obvious visible meaning and Bailey-Ross et al. (2019) observed an elaboration effect for figurative art that included symbolic elements when the context offered some insight into the meaning, rather than a commentary on the aesthetics of the image. Taken together these findings suggest that it is not simply the presence of contextual information, which is important, but rather whether the information is relevant for the artwork and the extent to which it helps the viewer understand the work.

The precise psychological mechanism by which providing contextual information leads to a more profound aesthetic experience remains controversial. One possibility is that the information allows the observer to direct their attention rapidly and efficiently to the most relevant elements of the artwork while ignoring the irrelevant elements. There is good evidence from eye tracking studies of scene perception that contextual information contributes to fast and efficient scene processing which are associated with fixations on the elements of the scene which are most meaningful (for a review see Võ, 2021). When applied to artworks, eye tracking has shown that participants tend to look longer at paintings they evaluate as beautiful (Jankowski, Francuz, Oleś, Chmielnicka-Kuter, & Augustynowicz, 2020) and to fixate first, for longer and more often on stimuli that they rated to be more aesthetically pleasing (Williams, McSorley, & McCloy, 2018). Another possibility could be related to processing fluency, which describes the ease with which an individual processes information. This concept proposed by Reber, Schwarz, and Winkielman (2004) suggests that aesthetic pleasure is influenced by the perceiver's processing dynamics, specifically the fluency with which an object can be processed, with greater fluency leading to a more positive aesthetic response. This proposal is supported by evidence that variables facilitating the processing of a stimulus (i.e., goodness of form, symmetry, figure-ground contrast, stimulus repetition, prototypicality, as well as perceptual and conceptual priming procedures) result in more positive affective reactions and more favorable judgments of preference.

Gaze behavior has also been found to differ depending on the information about the artwork provided to the perceiver. Kapoula, Daunys, Herbez, and Yang (2009) looked at the effect of title of cubist paintings on eye-movements patterns. Participants could either explore paintings without knowing the title, with a title they were asked to create themselves or the real title. They reported that fixation duration and saccade amplitudes were increased in the real title condition compared to the other condition. Similarly, Bailey-Ross et al. (2019) reported that contextual information directed gaze away from faces and towards symbolic elements of figurative works. On first inspection, these data seem consistent with the idea that contextual information drives efficient sampling of the most relevant elements in the scene, which in turn allows rapid understanding of the artwork and the increased aesthetic experience associated with transition from level 1 to level 2 of the 3-stage model. However, this conclusion may be somewhat premature because Kapoula et al. (2009) did not explicitly measure aesthetic appreciation, and Bailey-Ross et al. (2019) reported that contextually driven changes in gaze were associated with reduced rather than increased liking for the artwork.

To summarize, the three-level model from Bullot and Reber (2013) argues that contextual information enhances aesthetic experience when the meaning of an artwork is ambiguous. Eye tracking studies show that people fixate more and for longer on elements they rate more aesthetically pleasing, and there is convincing evidence that contextual information affects the distribution of gaze, particularly saccade amplitude and fixation duration. However, the evidence that these contextually triggered changes in oculomotor behavior^1^ are directly linked to changes in aesthetic experience is rather mixed. The goal of the current study was to test the claim that providing relevant contextual information will enhance the aesthetic experience of artworks whose meaning is ambiguous by changing the pattern of gaze. To this end we measured oculomotor behavior and aesthetic appreciation in 15 artworks representing figurative and 15 artworks representing abstract art. All paintings were chosen based on Tate's definition that Figurative art is “any form of modern art that retains strong reference to the real world and particularly to the human figure” and Abstract Art is “art that does not attempt to represent an accurate depiction of a visual reality, but instead use shapes, colors, forms and gestural marks to achieve its effect.”

It was predicted that providing contextual information (a) would increase aesthetic appreciation, (b) change the pattern of gaze, such that regions that are relevant to understanding the meaning of the artwork should receive more fixations which are of a longer duration and (c) that the effect of context on gaze and aesthetic appreciation should be greater for abstract artworks as the meaning of the artwork is ambiguous.

## Methods

### Participants

A total of 30 participants took part in the study. To determine the sample size, we ran as power analysis using G*power v3.1 (Faul, Erdfelder, Lang, & Buchner, 2007), based on the data collected in an online pilot study (see Supplementary Material) where we found an effect size of η^2^_p_ = 0.56 for the main effect of contextual information on aesthetic appreciation. Results of the analysis indicated that at least 29 participants would be required (80% power and α = 0.05). Participants’ ages ranged from 20 to 23 years old (*M*_age_ = 20.70, *SD*_age_ = 0.66). All had normal or corrected-to-normal vision, and none were color blind or held any qualifications in Art History. Participants were recruited either through the Durham University Psychology Department Participant Pool, receiving course credit for their participation, or through personal contact. This experiment was conducted following the BPS guidelines and was approved by the Durham University Ethics Committee.

### Apparatus

Stimulus presentation was controlled by Experiment Builder (SR Research Ltd., Mississauga, Ontario, Canada) on a 21″ CRT monitor with a resolution of 1024 by 768 pixels and a refresh rate of 100 Hz. The distance between the participant's eyes and the monitor was 55 cm and was kept constant by stabilizing the participant's head with a chin rest. Eye-position data of the right eye were recorded with a remote EyeLink 1000 system (SR Research Ltd., Mississauga, Ontario, Canada) with a sampling rate of 1000 Hz (accuracy: 0.5°; precision: 0.01° RMS). Vision was binocular.

### Material and measures

Thirty images were selected, 15 representing figurative and 15 representing abstract artwork (see Supplementary Table S1 in Supplementary Material for full list of artworks). Each artwork was preceded with contextual information that could be either (a) artist name, title, year, (b) artist name, title, year, and aesthetic information (i.e., a description of the artist's methods in creating the painting) or (c) artist name, title, year, and semantic information (i.e., a description of the artist's intentions for the meaning of the painting. This led to a total of six experimental conditions (2 artworks genre × 3 contextual information). All images and accompanying texts were sourced primarily from online information from reputable art galleries (e.g., Tate), the information provided by the artist themselves (i.e., personal website), or information provided at auctions (e.g., Sotheby's). All images were of high quality and set against a completely white background when presented to participants. We made sure that contextual information did not mix aesthetic and semantic content (see Appendix). Information was only provided on the screen before the corresponding painting (i.e., no contextual information was provided on the same screen as any painting—see procedure).

Aesthetic appreciation and understanding of the paintings were measured using five questions: (1) “I like this artwork,” (2) “I find this artwork interesting,” (3) “I felt able to understand the artwork,” (4) “I could get a sense of what the artist wanted to express,” and (5) “I am familiar with this painting.” All questions were adapted from Silvia (2005) and Swami (2013). Answers were coded using a seven-point Likert- type scale (e.g., 1 = strongly disagree to 7 = strongly agree) except for the familiarity ratings (1 = definitely not familiar, 5 = definitely familiar). We first calculated the average score separately for each question before combining them to produce the “Liking and Interest” and the “Understanding” score (see Data Selection and Analysis).

### Procedure

**Figure 1.**
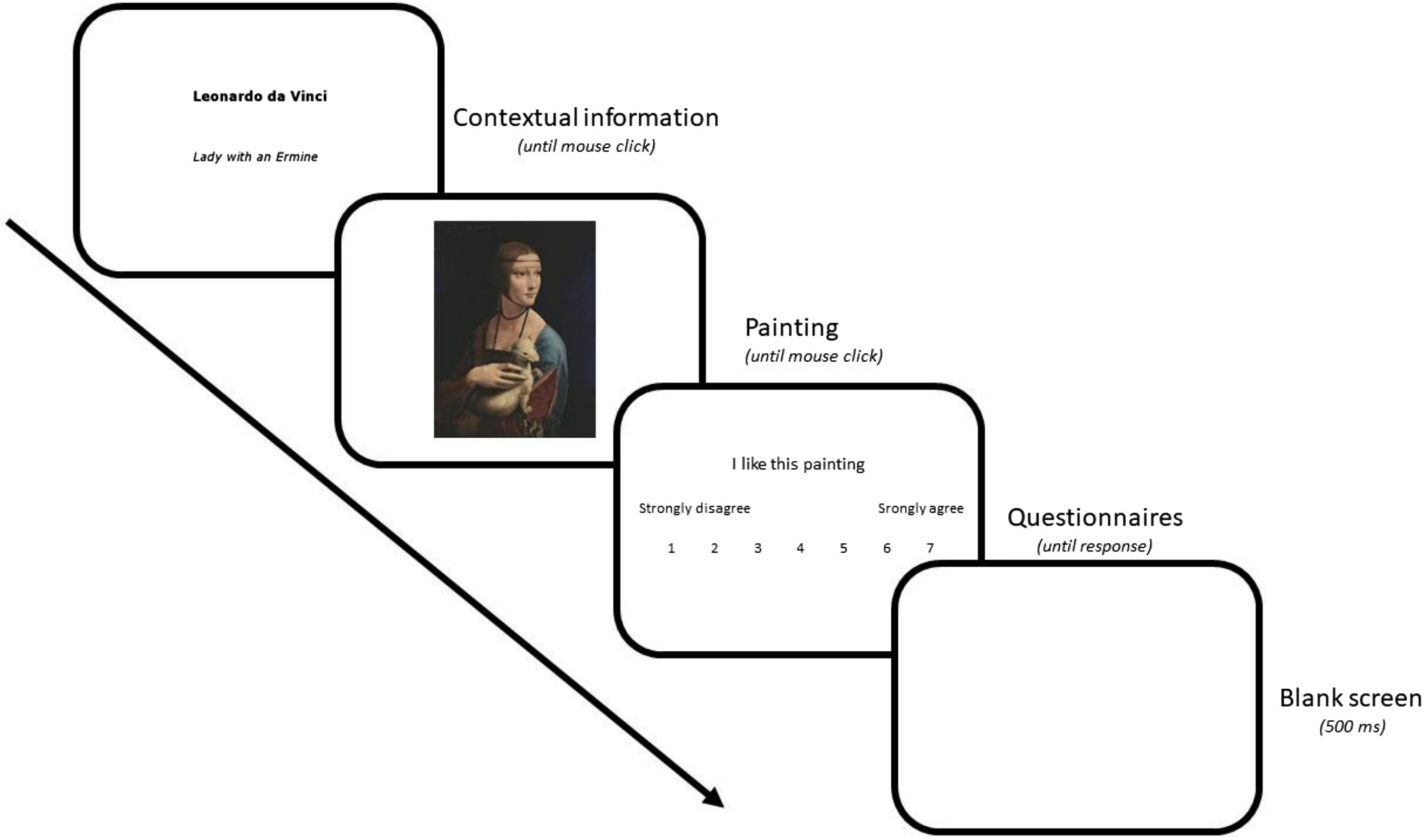
Example of trial sequence.

The room was dark except for a dim indirect light source, and the participant was seated in an adjustable chair in front of a computer screen. Trials started after setting up the eye tracker and running a five-point calibration phase. If the average difference between the dot location and the gaze position was satisfactory (less than 0.70°), the block of trials began. Otherwise, another calibration phase was performed. The trial then started with the contextual information for the participants to read before using a computer mouse click to see the corresponding painting. Participants could spend as long as they wanted to explore the paintings before clicking on the mouse to see the questions (see Figure 1). Participants were required to rate their liking, understanding, interest and familiarity of the artwork through forced-choice responses.

Participants’ instructions were as follows:

You are going to be presented with a series of artworks. Before each artwork, you will be presented with information about the art piece.

Please take time to read the information, once you've read it, click on the mouse to see the artwork. Take your time to study the piece, once you are done click on the mouse. You will be then presented with a series of questions regarding your appreciation, understanding and familiarity with each piece.

This was repeated for each of the 30 paintings with the varying levels of information and types of artworks. A five-second minimum time delay was placed on both the contextual information and the artwork to encourage participants to fully engage with the stimuli. Participants saw all the 30 images; the order of presentation was randomized between participants.

## Results

### Data selection and analysis

The median familiarity rating for our 30 paintings was of 1 (mean = 1.8); therefore we can be confident that most if not all our participants had limited experience with art. Three paintings received a median familiarity score of 5 (see Supplementary Table S2). These paintings were *Auto-portrait* by Vincent Van Gogh, *Composition* by Piet Mondrian, and *A Sunday Afternoon on the Island of La Grande Jatte* from George Seurat. We ran the analysis both with and without these three paintings (see Supplementary Material); however, because the main results did not significantly differ between the two sets of analysis, these three artworks were retained for the final data analysis.

The first set of analysis were performed on the average ratings. Following Swami's (2013) methodology we combined the results for liking and interest ratings to produce the “Liking and Interest” mean score for each participant in each condition. Similarly, the results of the two ratings regarding the understanding of the painting and the artist's intention were combined to form the “Understanding” average score. Note that contrary to Swami (2013) we decided not to label the combined liking and interest ratings as “aesthetic appreciation” as this study emphasizes on the fact that understanding is also a form of appreciation.

Saccades/fixations were detected using the built-in EyeLink detection algorithm using the standard velocity, acceleration, and displacement thresholds of 30°/s, 8,000°/s,^2^ and 0.1°. Fixations from the onset of the artwork and the mouse click were analyzed and only fixations longer than 100 ms were retained.

**Figure 2.**
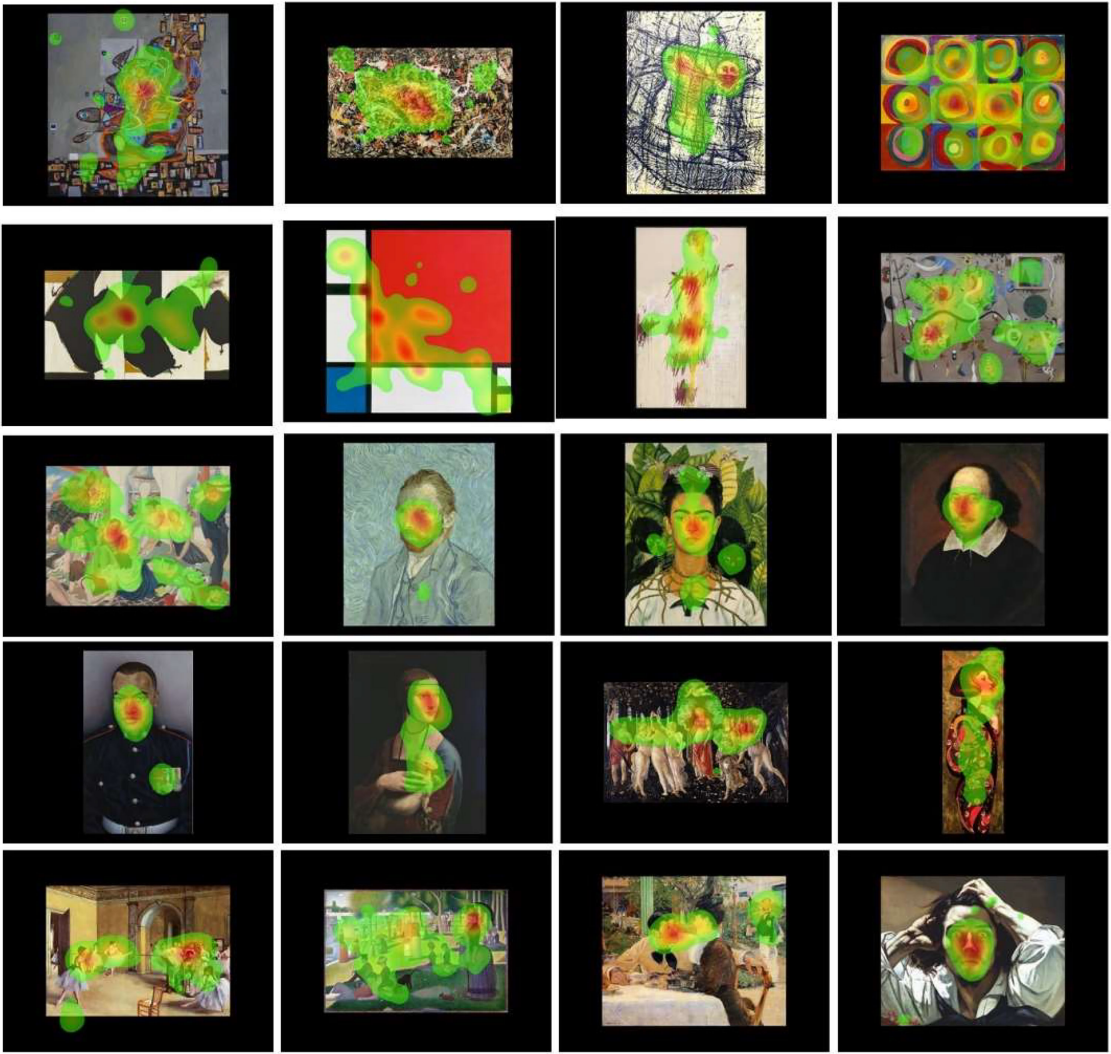
Examples of heatmaps showing the distribution of gaze across each artwork in the three contextual information conditions for abstract (two upper rows) and figurative (three lower rows) art. List of depicted artworks authors, titles, year, copyrights, and sources: Gheorghe Virtosu, *Master of the Waves*, 2017 (VIRTOSU ART, source: Virtosu Art Gallery); Jackson Pollock, *Convergence*, 1952 (The Pollock-Krasner Foundation ARS, NY and DACS, London 2024, source: Wikiart); Max Ernst, *Composition (Un Chant d'Amour)*, 1958 (ADAGP, Paris and DACS, London 2024, source: Art Institute Chicago); Wassily Kandinsky, *Colour Study Squares With Concentric Circles*, 1913 (public domain, source: Wikimedia Commons); Robert Motherwell, *Elegy to the Spanish Republic*, 1965 (The Solomon R. Guggenheim Foundation/Art Resource, NY/ Scala, Florence, Source: Scala Group); Piet Mondrian, *Composition II in Red, Blue, and Yellow*, 1930 (public domain, source: Wikimedia Commons); Cy Twombly, *Primavera*, 1993–1995 (Cy Twombly Foundation, source: Tate); Joan Miró, *Harlequin's Carnival*, 1924–1925 (Successió Miró/ADAGP, Paris and DACS London 2024, source: Wikiart); Mary Adshead, *The Cruise*, 1934 (Estate of Mary Adshead. All Rights Reserved, DACS 2024, source: Tate); Vincent van Gogh, *Self-Portrait*, 1889 (public domain, source: Wikimedia Commons); Frida Kahlo, *Self-Portrait With Thorn Necklace and Hummingbird*, 1940 (Banco de México Diego Rivera Frida Kahlo Museums Trust, Mexico, D.F./DACS 2024, source: Wikipedia); Portrait by unknown artist, *The Chandos*, (1600-1610) (public domain, source: Wikimedia Commons); Emma Wesley, *Johnson Gideon Beharry*, 2006 (National Portrait Gallery, London, source: National Portrait Gallery); Leonardo da Vinci, *Lady With an Ermine*, 1489–1491 (public domain, source: Wikimedia Commons); Botticelli, *Primavera*, 1470–1980 (public domain, source: Wikimedia Commons); Lauren Brevner, *Iris*, 2015 (Lauren Brevner, source: Lauren Brevner); Edgar Degas, *Dance Class at the Opera*, 1872 (public domain, source: Wikimedia Commons); George Seurat, *A Sunday Afternoon on the Island of La Grande Jatte*, 1884–1886 (public domain, source: Wikimedia Commons); Édouard Manet, *Chez le Père Lathuille (At the Père Lathuille Restaurant)*, 1879 (public domain, source: Wikimedia Commons); Gustave Courbet, *Le Désespéré (The Desperate Man)*, (1843–1845) (public domain, source: Wikimedia Commons).

For the gaze-behavior we first analyzed the total time participants spent exploring each artwork. We then identified regions of interests (ROIs) and analyzed the number of fixation and the duration of fixations on each ROIs. To define the ROIs, we analyzed the data of six randomly chosen participants and looked at the fixation maps of each painting^2^ (see Figure 2). Using the spatial overlay, we coded ROIs manually using the fixation maps as a canvas. Note that the data of these six participants were not included in the final data set.

All measures (ratings and gaze behavior) were analyzed using a 2 (artistic genre: figurative or abstract) × 3 (contextual information: title, aesthetic, semantic) repeated measures analysis of variance (ANOVA). For post-hoc analysis we used the Bonferroni method for multiple comparisons. In cases where Mauchly's test of sphericity indicated a violation of the assumption of sphericity we used the Greenhouse-Geisser correction. All descriptive and inferential statistics were performed using JASP 0.16.3 (2022).

### Liking and interest

**Figure 3.**
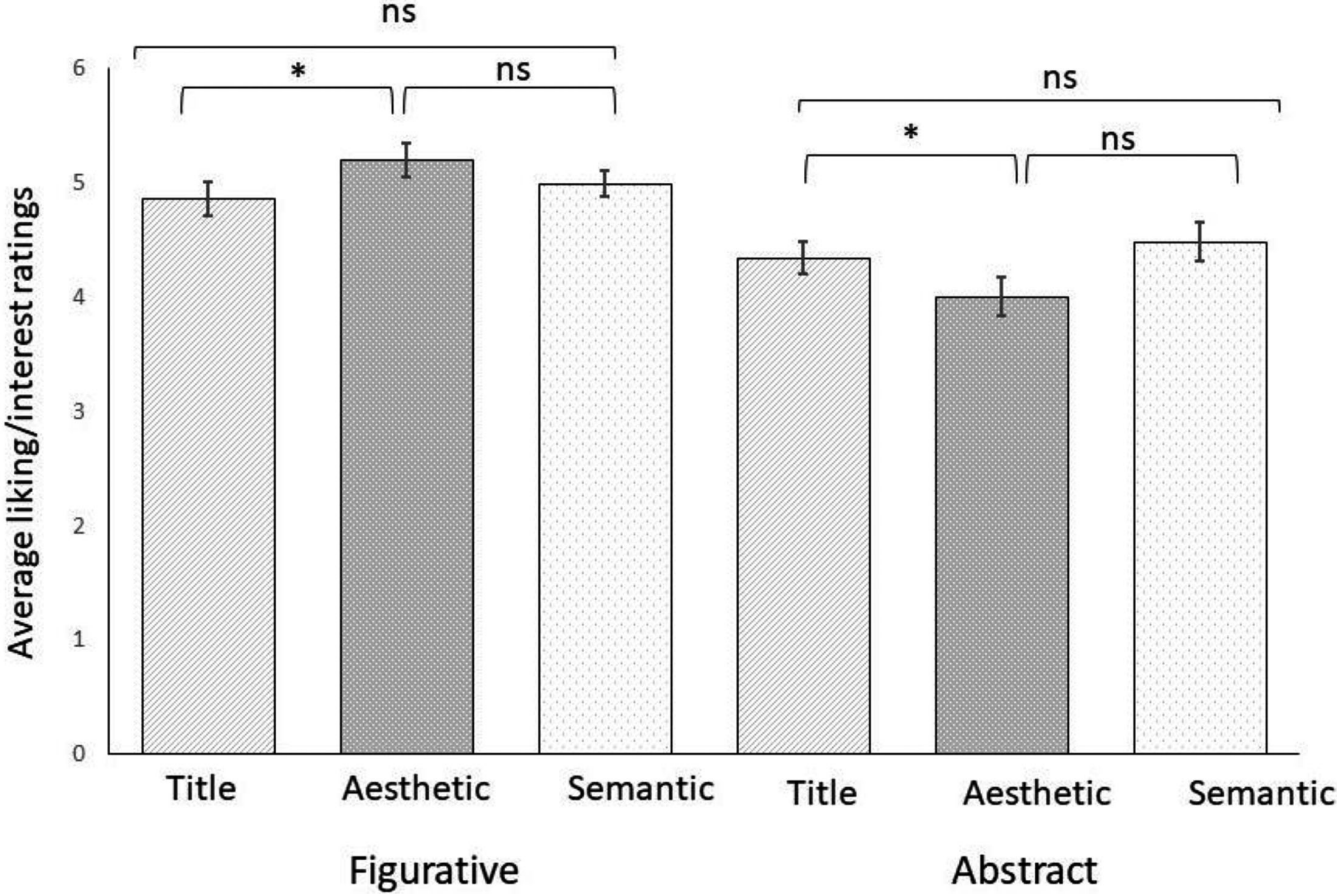
Average liking/interest ratings as a function of the type of contextual information (title, aesthetic, semantic) separately for figurative and abstract artworks. *Note*: Error bars show 95% confidence intervals: * *p* < 0.05; ** *p* < 0.01; *** *p* < 0.001.

Results of the ANOVA revealed a significant main effect of artistic genre (*F*(1, 29) = 24.67, *p* < 0.001, η^2^_p_ = 0.46), participants tended to give higher ratings to Figurative painting (*M* = 5.01, *SD* = 0.70) compared to Abstract paintings (*M* = 4.64, *SD* = 0.64). There was no significant main effect of context (*F*(1, 58) = 1.17, *p* = 0.316, η^2^_p_ = 0.04), but there was a significant interaction of artistic genre × context (*F*(2, 58) = 8.57, *p* < 0.001, η^2^_p_ = 0.23) (see Figure 3).

The significant artistic genre × context interaction was broken down using two one-way ANOVAs. For figurative artworks, there was a significant main effect of contextual information on liking (*F*(2, 58) = 4.19, *p* = 0.020, η^2^ = 0.13). Post-hoc t-tests revealed that participants gave significantly higher liking ratings in the aesthetic condition (*M* = 5.20, *SD* = 0.86) compared to the title only condition (*M* = 4.86, *SD* = 0.85; *t*(29) = 2.87, *p* = 0.017, *d* = 0.42) but not compared to the semantic condition (*M* = 4.99, *SD* = 0.66, *p* = 0.237). The title only and semantic condition were not significantly different (*p* > 0.99). For the abstract artworks there was a significant effect of contextual information on liking/interest ratings (*F*(2, 58) = 4.94, *p* = 0.010, η^2^ = 0.15). Post-hoc t-test showed that, contrary to figurative artworks, ratings were lowest in the aesthetic condition (*M* = 4, *SD = 1*). The difference was statistically significant compared to the semantic condition (*M* = 4.48, *SD* = 1; *t*(29) = 3.06, *p* = 0.010, *d* = 0.47), but not the title-only condition (*M* = 4.34, *SD* = 0.84, *p* = 0.106). There was no difference between title and semantic conditions (*p* > 0.99).

### Understanding

**Figure 4.**
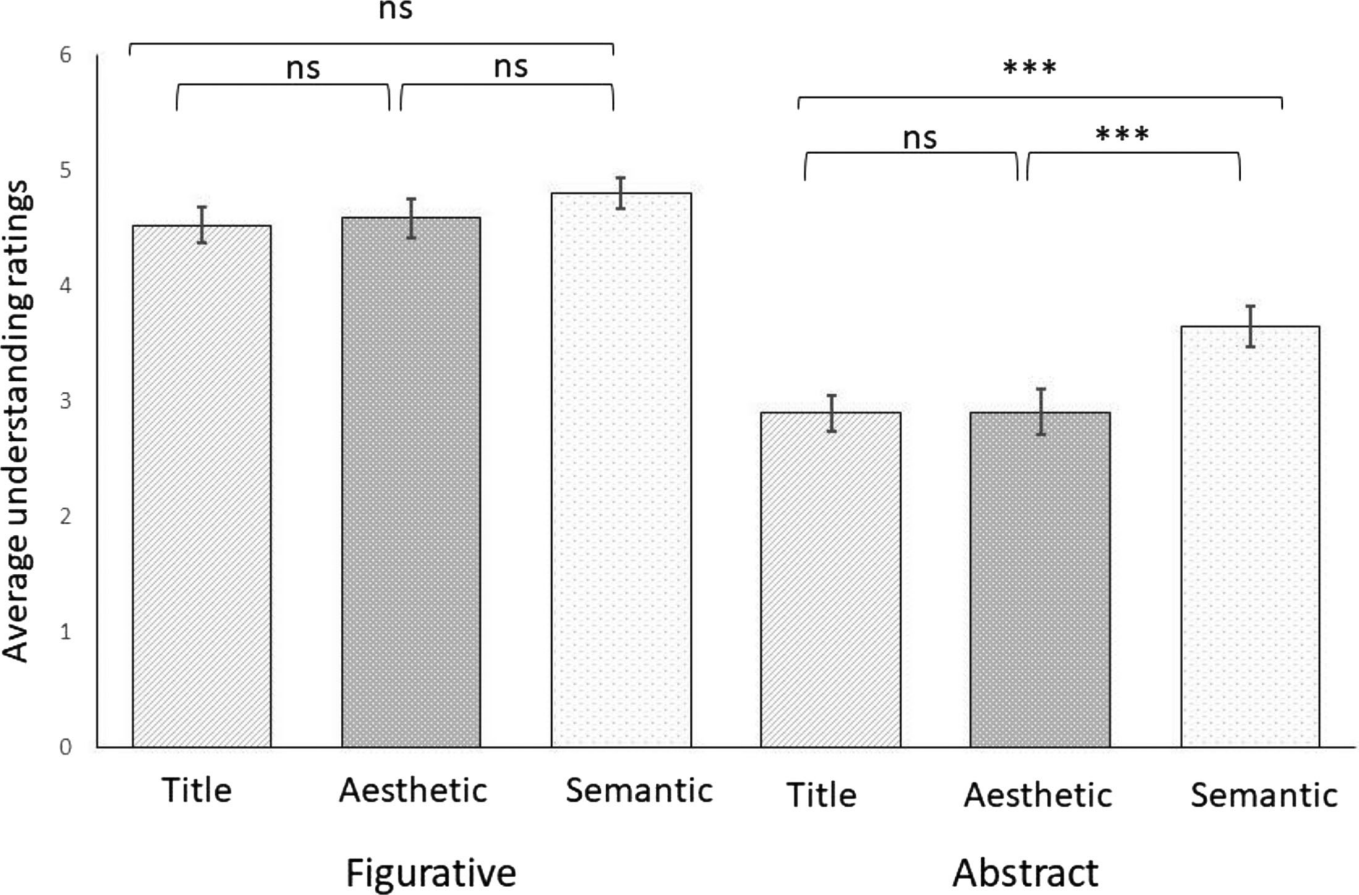
Average understanding ratings as a function of the type of contextual information (title, aesthetic, semantic) separately for figurative and abstract artworks. *Note:* Error bars show 95% confidence intervals: * *p* < 0.05; ** *p* < 0.01; *** *p* < 0.001.

ANOVA revealed significant effects of artistic genre (*F*(1, 29) = 94.09, *p* < 0.001, η^2^_p_ = 0.76) such that participants gave higher ratings to the figurative artworks (*M* = 4.65, *SD* = 0.83) compared to the abstract artworks (*M* = 3.15, *SD* = 0.95) and of context (*F*(2, 58) = 23.32, *p* < 0.001, η^2^_p_ = 0.45), and a significant interaction effect between context × artistic genre (*F*(2, 58) = 4.6, *p* = 0.014, η^2^_p_ = 0.14) (see Figure 4). When breaking down the significant interaction using one-way ANOVAs separately for each genre, we found that for figurative artworks there was small but significant difference between the different contexts on understanding (*F*(2, 58) = 3.26, *p* = 0.046, η^2^ = 0.10), and Bonferroni corrected post-hoc *t*-test revealed no significant differences between the conditions (all *p* ≥ 0.055). For abstract artworks there was large and significant effect of contextual information on understanding (*F*(2, 58) = 19.76, *p* < 0.001, η^2^= 0.41). Participants gave higher understanding ratings in the semantic condition (*M* = 3.65, *SD* = 1.05) compared to the aesthetic condition (*M* = 2.91, *SD* = 1.14, *t*(29) = 5.38, *p* < 0.001, *d* = 0.71) and the title condition (*M* = 2.90, *SD* = 0.92, *t*(29) = 5.50, *p* < 0.001, *d* = 0.72). The difference in ratings between the aesthetic and the title conditions was not statistically significant (*p* > 0.99).

### Total exploration duration

**Figure 5.**
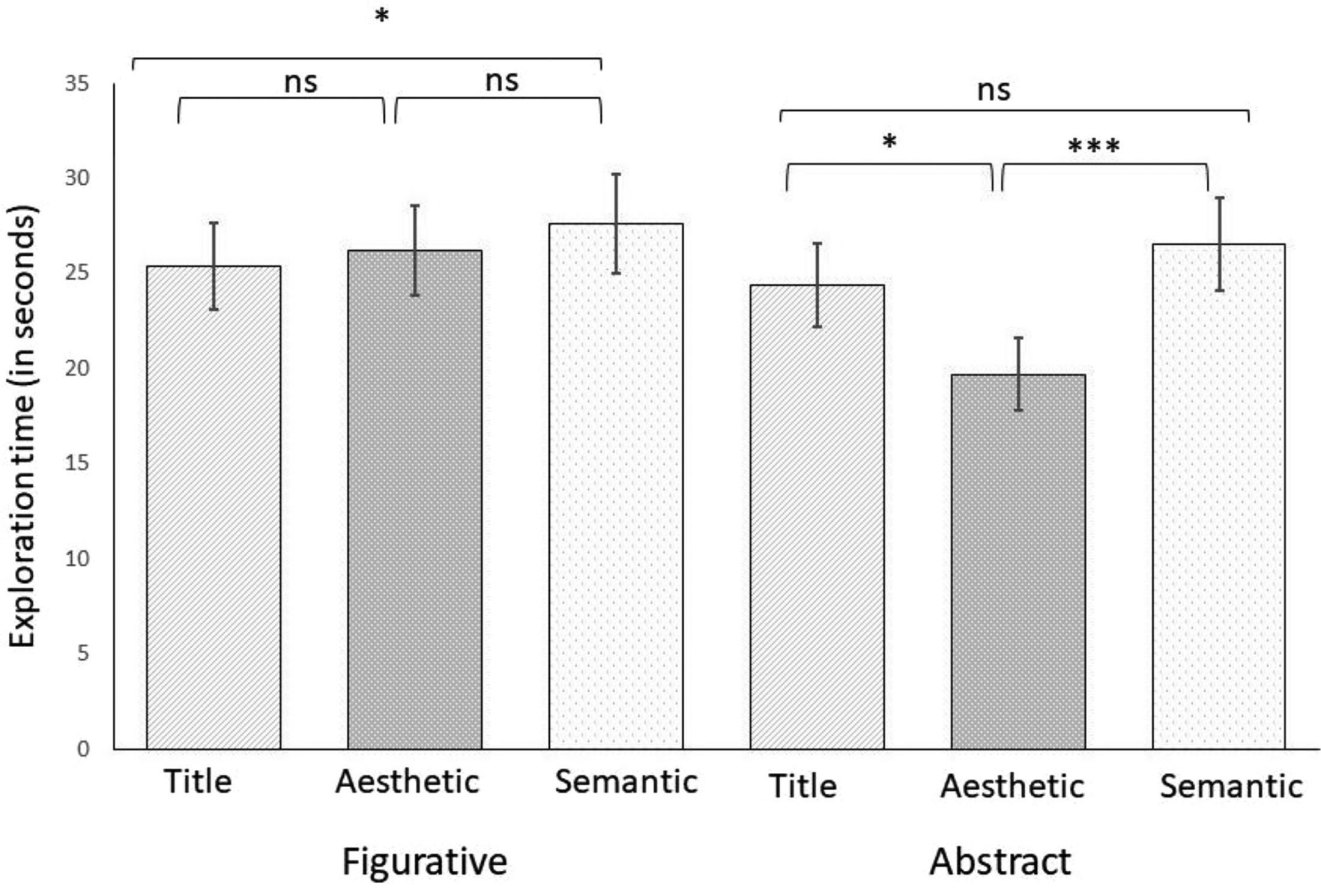
Exploration time as a function of the type of contextual information (title, aesthetic, semantic) separately for figurative and abstract artworks. *Note*: Error bars show 95% confidence intervals: * *p* < 0.05; ** *p* < 0.01; *** *p* < 0.001.

Exploration duration was measured as the median time (in seconds) participants spend looking at each of the artworks. We found a significant effect of artistic genre (*F*(1, 29) = 9.80, *p* = 0.004, η^2^_p_ = 0.25) , a significant effect of contextual information (*F*(2, 58) = 6.72, *p* = 0.002, η^2^_p_ = 0.19), as well as a significant interaction (*F*(1, 29) = 7.89, *p* < 0.001, η^2^_p_ = 0.21) that was broken down using two one-way ANOVAs. For figurative artworks we found significant differences in the exploration duration between the three information conditions (*F*(2, 58) = 3.68, *p* = 0.031, η^2^ = 0.11) (see Figure 5). Bonferroni corrected post-hoc *t*-test showed that participants’ median exploration time (in seconds) was significantly longer in the semantic condition (*M* = 26.27, *SD* = 14.14) compared to the titular condition (*M* = 23.27, *SD* = 13.04, *t*(29) = 2.55, *p* = 0.040, *d* = 0.47), but not compared to the aesthetic condition (*M* = 26.27, *SD* = 14.14, *p* > 0.99). There was also no significant difference between the titular condition and the aesthetic condition (*p* = 0.129). We also found a significant effect of the contextual information on median exploration time for the abstract artwork (*F*(1, 58) = 9.15, *p* < 0.001, η^2^ = 0.24). Again, participants spent significantly more time exploring abstract paintings when they were preceded with semantic information (*M* = 25.44, *SD* = 14.67), and this was significantly longer compared to the aesthetic condition (*M* = 18.85, *SD* = 11.24) (*t*(29) = 4.25, *p* < 0.001, *d* = 0.76) but not compared to the title condition (*M* = 22.86, *SD* = 12.71, *p* = 0.303). Participants also tended to spend less time exploring paintings in the aesthetic condition compared to the titular condition (*t*(29) = 2.58, *p* = 0.037, *d* = 0.47).

### Proportion of fixations on relevant ROIs

Relevant ROIs were defined based on fixations maps of six participants that were not included in the final sample size (see Data Selection and Analysis). Fixations maps for the 30 paintings for the final sample (*N* = 30) are presented in the Supplementary Figure S3. Proportion of fixation on relevant ROIs were calculated by dividing the number of fixations falling in the ROIs by the total number of fixations on the artwork. These proportions were area-normalized by dividing the number of fixations within the ROIs by its area (Smilek, Birmingham, Cameron, Bischof, & Kingstone, 2006). The 2 × 3 ANOVA was then run on the median number of fixations.

**Figure 6.**
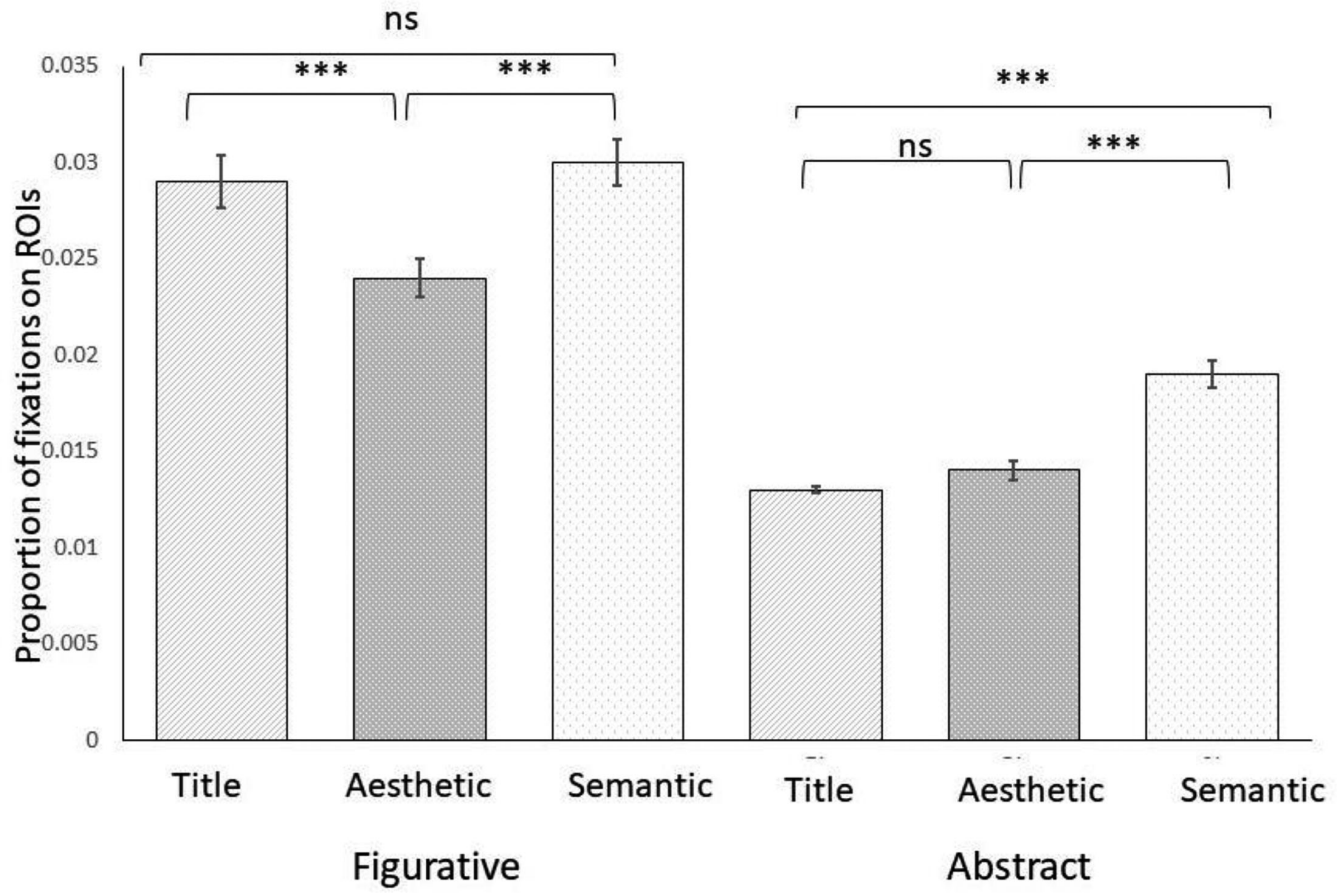
Proportion of fixations on relevant ROIs as a function of the type of contextual information (title, aesthetic, semantic) separately for figurative and abstract artworks. *Note*: Error bars show 95% confidence intervals:* *p* < 0.05; ** *p* < 0.01; *** *p* < 0.001.

We found a significant main effect of artistic genre (*F*(1, 29) = 188.17, *p* < 0.001, η^2^_p_ = 0.87), a significant main effect of contextual information (*F*(2, 58) = 25.43, *p* < 0.001, η^2^_p_ = 0.47), as well as a significant interaction between contextual information × artistic genre (*F*(2, 58) = 10.55, *p* < 0.001, η^2^_p_ = 0.27). Contextual information had a significant effect on the proportion of fixations within the relevant ROIs in both figurative (*F*(2, 58) = 11.16, *p* < 0.001, η^2^ = 0.28) and abstract (*F*(2, 58) = 54.49, *p* < 0.001, η^2^ = 0.65) artistic genre. As can be seen on Figure 6, for figurative artworks the normalized proportion of fixations made within the relevant ROIs was lower in the aesthetic condition (*M* = 0.024, *SD* = 0.006) compared to the title (*M* = 0.029, *SD* = 0.008) and semantic conditions (*M* = 0.030, *SD* = 0.007). Post-hoc *t*-testing revealed that the differences were statistically significant (*t*(29) = 3.99, *p* < 0.001, *d* = 0.73, *t*(29) = 4.19, *p* < 0.001, *d* = 0.77 for title and semantic condition, respectively). The difference in the normalized proportion of fixation between title and semantic conditions was not statistically significant (*p* > 0.99).

In the abstract condition, proportions of fixations on relevant ROIs were greater in the semantic condition (*M* = 0.019, *SD* = 0.004), and this was significantly greater compared to the aesthetic condition (*M* = 0.014, *SD* = 0.003, *t*(29) = 8.54, *p* < 0.001, *d* = 1.56) and the title condition (*M* = 0.013, *SD* = 0.001, *t*(29) = 9.48, *p* < 0.001, *d* = 1.73). The difference between aesthetic and title conditions was not significant (*p* > 0.99).

### Fixation duration on relevant ROIs

**Figure 7.**
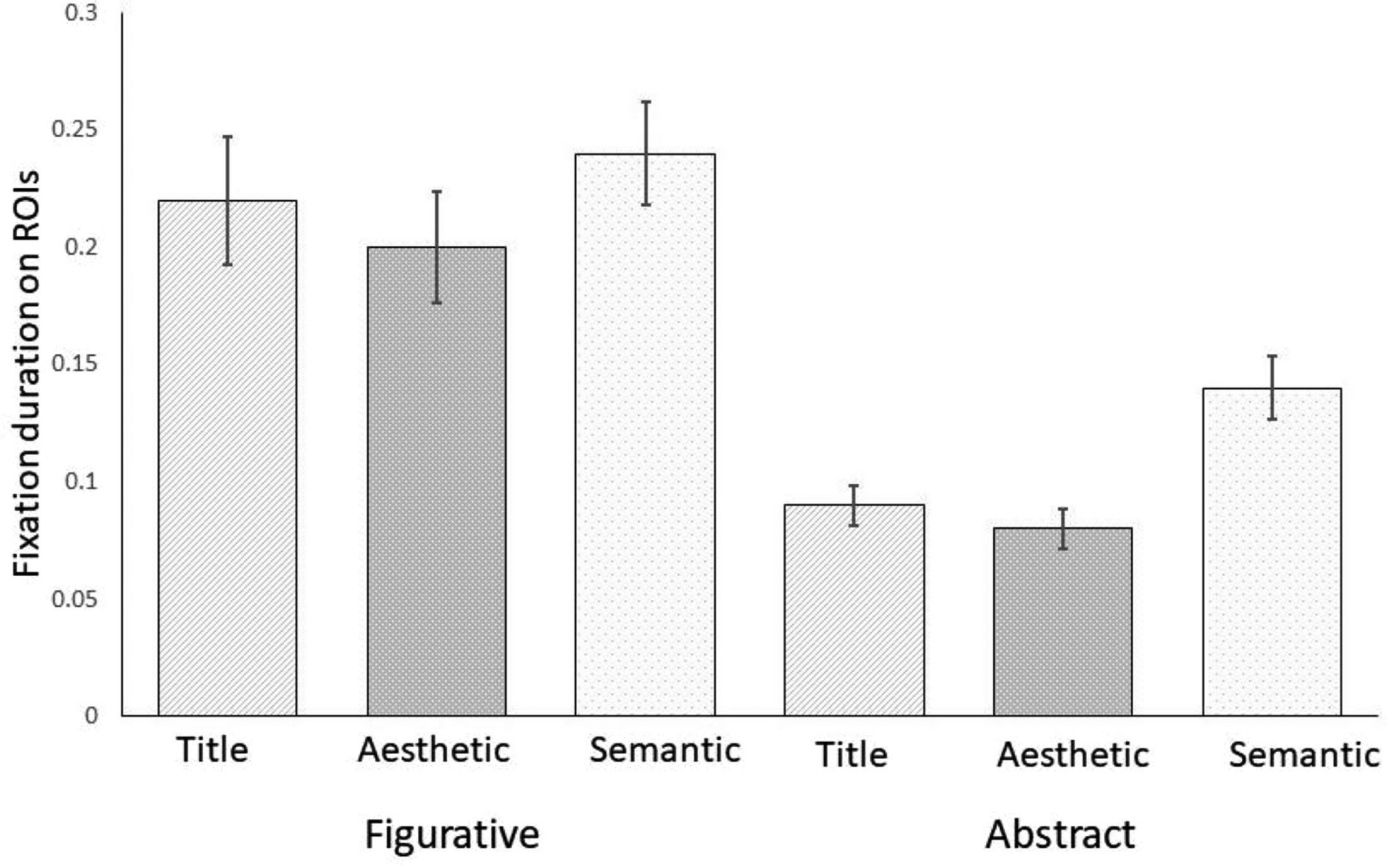
Fixation durations on relevant ROIs as a function of the type of contextual information (title, aesthetic, semantic) separately for figurative and abstract artworks. *Note:* Error bars show 95% confidence intervals.

Median fixation duration on relevant ROIs were also area-normalized using the same calculation as described above. Results of the ANOVA revealed a significant main effect of artwork on median normalized fixation duration (*F*(1, 29) = 58.44, *p* < 0.001, η^2^_p_ = 0.41), as well as a significant main effect of contextual information (*F*(2, 58) = 15.60, *p* < 0.001, η^2^_p_ = 0.06). Normalized median fixation durations were longer in the semantic condition (*M* = 0.19, *SD* = 0.10) compared to title (*M* = 0.15, *SD* = 0.09) and aesthetic (*M* = 0.14, *SD* = 0.09). There were significant differences between semantic and titular (*t*(29) = 4.11, *p* < 0.001, *d* = 0.75) and between semantic and (*t*(29) = 5.33, *p* < 0.001, *d* = 0.97), but not between title and design (*p* = 0.682). However, the interaction between artwork and aesthetic condition was not significant (*F*(2, 58) = 0.84, *p* = 0.438, n^2^_p_ = 0.03) (see Figure 7).

## Discussion

The aim of the present study was to investigate whether contextual information has an effect on the appreciation of artworks, specifically regarding the figurative and abstract art genres, in reference to Bullot and Reber's (2013) psychohistorical framework. Overall, our results show that contextual information did affect appreciation of artworks as compared to titular information alone, and semantic contextual information not only increased in total exploration time but also increased the number of fixations and the median fixation duration on relevant ROIs. However, this effect was not systematically observed for aesthetic contextual information.

Regardless of contextual information, results of the current study show that figurative artworks received significantly higher liking/ interest and understanding ratings than abstract artworks, which support previous findings (e.g., Castellotti et al., 2023; Mastandrea, Fagioli, & Biasi, 2019; Moore & West, 2012; Swami, 2013). This effect was dependent on the type of contextual information provided to the observer. Notably, the aesthetic context produced highest liking/interest ratings for figurative art, but lowest ratings for the abstract art. Consistent with Swami (2013), the semantic context produced higher liking ratings in the abstract art condition, consistent with the claim that knowing the artist's intention leads to greater appreciation (Darda & Chatterjee, 2023; Davies, 2013; Iosifyan, 2021; Jacquette, 2014). However, the observation that aesthetic contextual information did not increase both liking/interest and understanding compared to the title only condition in abstract art suggests that contextual information does not always have a positive effect on appreciation of artworks, contrary to the broader idea that contextual information always increases appreciation regardless of genre, as suggested by Bullot and Reber's (2013).

In line with aesthetic appreciation, gaze behavior analysis showed that participants tend to spend more time, have more fixations and longer fixations within relevant ROIs when exploring figurative compared to abstract artwork, regardless of the contextual information. This is consistent with previous studies that have shown that people tend to explore for longer period artwork that they found aesthetically pleasing (Brieber, Nadal, Leder, & Rosenberg, 2014; Jankowski et al., 2020; Williams, et al., 2018). More interestingly, we found that semantic information had no effect on the proportion of fixations on relevant ROIs. Because figurative artwork often portrays recognizable objects, scenes, or figures, the addition of aesthetic information might not be needed as a “guide” to explore and understand the artwork. We also found that the proportion of fixations on what was deemed as “relevant” ROIs were lower in the aesthetic condition. Work from Bailey-Ross et al. (2019) found that aesthetic, contextual information provided with artworks tend to redirect the gaze away from faces. Given that our ROIs were centered around faces areas (see Figure 2), it might be the case that aesthetic information drew participant's gaze away from faces in our study as well.

For abstract artwork, we found an increase in total exploration time, number of fixations and length of fixations in the semantic condition compared to both title and aesthetic condition, which is congruent with our hypothesis. The observation that the proportion of fixations were greater on ‘relevant’ ROIs in the semantic condition suggest that fixations are directed towards semantically meaningful features. Top-down cognitive processes can indeed guide fixation selection throughout the course of viewing, as shown by Nyström and Holmqvist (2008) who reported that, in scene viewing, participant tend to fixate more on meaningful stimuli. However, this was not the case for aesthetic contextual information as we found lower exploration times and no differences between title and aesthetic information for ROIs analysis. On first inspection this finding appears problematic for the hypothesis that looking time would be greater for abstract artworks in both the aesthetic and semantic conditions (Leder, Carbon, & Ripsas, 2006). It might be the case that providing information about the artist's intentions may constrain participant's imagination and limit the need for prolonged exploration. Indeed, by providing the aesthetic context the viewer knows where they should look but remains unsure about what the painting means (unlike in the semantic context).

Although our study did not show any effect of aesthetic contextual information on gaze behavior, we observed an increase in exploration time, number of fixations and fixation duration in the semantic contextual information condition. Abstract art often depicts complex visual stimuli that may require more time to process and understand as there are no clear and recognizable objects or subjects (Leder, Belke, Oeberst, & Augustin, 2004). Drawing on the principles of Gestalt theory (Koffka, 2013), we may interpret the observation of longer fixations on relevant parts of the abstract composition as a possible way to make sense of the overall visual structure and relationships between elements (Behrens, 1998). Abstract artworks are designed to disrupt automatic processing, making them inherently ambiguous without contextual information whereas a hallmark of good figurative artworks is that they clearly display a real-life scene. Therefore, guided by the semantic information provided, participants may have fixated longer on different elements within the abstract artwork to explore its intricacies and ambiguous patterns. It is worth noting that by using already available contextual information (see methods) we were not able to systematically control for variability in the text provided. However, there is trade-off between reliability, where highly controlled contexts are used, and validity, where the actual words used in the gallery/websites are presented. For example, Bailey-Ross et al. (2019) created their own labels for the aesthetic context condition and found a significant effect of this contextual information on gaze behavior. Therefore, in the current study, it could have been the case that using created, less valid, contextual information would have led to a different pattern of gaze behavior.

Overall, our study seems to support Bullot and Reber's (2013) suggestion that the addition of semantic contextual information for abstract artwork might help participants to adopt an *artistic understanding* (as shown by greater understanding and greater proportion of fixations on relevant elements of the artworks). In our study, the titles of the artworks were quite informative, or at least sufficiently enough for the participants to adopt an *artistic design* stance. The fact that we did not observe any difference between titular and aesthetic condition might be explained by the fact that these types of contexts are not sufficient to give participants enough information to go from the *artistic design stance* to the *artistic understanding level*. However, contrary to what Bullot and Reber's (2013) model suggests, when it comes to figurative artwork the addition of contextual information was not necessary to have aesthetically pleasing experience. Even though on average participants spend more time looking at figurative paintings, this can be explained by aesthetic preference in general. At first inspection, the fact that participants explored figurative art for longer might seem to in contradiction with the notion of “processing fluency,” or the ease with which we process artwork related information, proposed by Reber, Schwarz, and Winkielman (2004). Indeed, if it is accepted that dwell time is influenced by the cognitive demands of a fixation, the “processing fluency” would seem to predict a negative relationship between fixation duration and enjoyment. More specifically, figurative artworks are thought to be easy-to-process stimuli; therefore they should be processed more quickly and so should require shorter fixations. However, we have found that the aesthetic context reduces the proportion of fixations on the ROIs; the same trend was observed for average fixation duration on ROIs (although not significant), but increased liking judgements is consistent with this idea. However, it is important to note that this effect was not observed for abstract art.

This relationship between figurative artwork and general pleasantness reflects a broader criticism of aesthetics research in general which draws upon early understandings of art such as Kant's (1952) notion that art is tightly coupled with beauty. In the late nineteenth and early twentieth century, artists such as Marcel Duchamp demonstrated that art did not inherently need to be beautiful so long as it allowed artists to be expressive and encouraged contemplation in the viewer (Danto, 2003). It is worth noting that most of the figurative artworks presented in the current study were depicting faces. Indeed, previous findings have shown that faces depicted in artworks play a very important role in aesthetic judgement (Massaro et al., 2012; Ro, Friggel, & Lavie, 2007; Villani et al., 2015), as well as in visual exploration (Bailey-Ross et al., 2019; Massaro et. al, 2012; Yarbus, 2013). Faces are crucial cues in our environment, frequently drawing our attention away from other elements within the same environment (Bindemann, Burton, Hooge, Jenkins, & de Haan, 2005). The human brain processes faces in a specialized manner, using distinct visual processing mechanisms that differ from those typically used in object recognition (Burton, Bruce, & Hancock, 1999), and numerous studies have uncovered face-selective brain regions, such as the fusiform face are (FFA) (Kanwisher, McDermott, & Chun, 1997). Studies have also demonstrated that faces are processed faster than other objects or even other body parts (e.g., Mouchetant-Rostaing, Giard, Bentin, Aguera, & Pernier, 2000). Crouzet, Kirchner, and Thorpe (2010) have shown that when a face and another image are presented on opposite sides of the fixation point, saccades can be triggered in the direction of the face in less than 100 ms. This motor response implies that the perceptual decision must have been made before 80 ms after stimulus presentation. These very early saccades in the direction of the face are triggered even when participants were specifically instructed that the saccade target was the other image, in other words, gaze attraction to faces is automatic or pre-attentive/pop out. Besides, in the current study, paintings were presented digitally on a computer screen, and previous studies have reported differences in visual exploration between museum context and digital context. For example, Quiroga et al. (2011) recorded eye-movement data of participants exploring Millai's Ophelia either in the context of the Tate Britain gallery or looking the digital image of the same artwork in the laboratory. They compared fixations on two regions of interest, either on the whole figure of Ophelia or on the rest of the painting. Interestingly, they found that overall participants who looked at the digital image did fixate more upon the figure of Ophelia, whereas participants in the museum made more fixations in the painting surrounding her, suggesting that the bias towards faces may be even larger when digital images are used. Therefore, it is not surprising that in our study, the identified ROIs for the figurative artwork presented are mostly localized on the face region, which could be seen as a bias. However, our approach here was more about whether contextual information changes these gaze patterns rather than which features in a painting, and therefore whether face saliency, influences fixation behavior. Nevertheless, it might be possible that when participants are presented with figurative artwork that do not depict faces, the effect of context might be closer to the one observed in the abstract genre. Indeed, if, as we have shown in the current study, contextual information is secondary to understanding paintings depicting faces, they may be more important in the case of artwork representing landscapes or still life. Having the information about what place the painting is depicting for example, or the process used by the artist to render a scene can change not only aesthetic preferences (e.g., Darda & Chatterjee, 2023) but also visual exploration (e.g., Villani et al., 2015). Therefore future studies may want to further look at the effects of contextual information on visual exploration using a wider range of figurative artwork, and more specifically paintings that do not depict mostly face(s).

Whilst not explicitly measured, it can be assumed that most of our participants were from Western cultures who consequently have a Western understanding of art. This limitation can be linked to the wider criticism of theories of aesthetic appreciation which focus on understanding the artist's specific intentions which is restricted to mainly Western interpretations of Western Fine Art. If we look at Islamic art for instance, one of the major features is repetitive geometric and floral/vegetal deigns called arabesques. The arabesques are thought to symbolize the transcendent and infinite nature Allah (Madden, 1975), therefore, natives from the culture of these artworks may not have the same motivation as Western viewers to understand what artists intended. That is because artists in this genre usually have common goals of using art for decoration and praising Allah, rather than having unique motivations. In Asian art, the traditional Chinese painter aims to capture not only the outer appearance of a subject but its inner essence as well its energy or spirit (Hearn, 2008). Therefore the extent to which conclusions based on individuals with a Western conceptualization of Western fine art generalize to other cultures remains an empirical question.

In conclusion, the present study demonstrated that contextual information influenced aesthetic appreciation and gaze behavior. Specifically, aesthetic contexts increased appreciation for figurative art but decreased appreciation for abstract art, whereas sematic contexts increased understanding for abstract art but not figurative art. These changes in appreciation were associated with changes in visual exploration behavior, such that the aesthetic context reduced overall looking time for abstract art, and the semantic context was associated with more frequent and longer duration fixations on salient regions of the artworks. These data are broadly consistent with Bullot and Reber's (2013) suggestion contextual information might help participants to adopt an *artistic understanding,* particularly for abstract art. However, contrary to Bullot and Reber (2013), contextual information did not enhance the aesthetic enjoyment of figurative art. These results demonstrate the importance of considering artistic style when examining the effect of contextual information on the exploration and enjoyment of artworks.

## Supplementary Material

Supplement 1

## Footnotes

1 We refer here to fixations (i.e., a period during which the eye is relatively still**)**

2 We decided not to draw ROIs based on *contextual* information because it would have limited the analysis to comparing semantic versus aesthetic context because in the title-only context, there was not sufficient information for us to draw meaningful ROIs. Therefore, to compare the three conditions, we opted to create ROIs based on a more realistic approach by examining participants' gaze. This method allows us to draw ROIs representing the most prominent parts of the artworks presented, whether they are figurative or abstract paintings.
