# Supplementary material for: How does contextual information affect aesthetic appreciation and gaze behavior in figurative and abstract artwork?
Source: J Vis. 2024 Nov 8;24(12):8. doi: 10.1167/jov.24.12.8 (PMC11552055; doi:10.1167/jov.24.12.8)
Supplement: Supplement 1 [file jovi-24-12-8_s001.docx]

**Supplementary material**

**Table 1**

*List of artworks presented and accompanying contextual information.*

(F = Figurative, A = Abstract, T = Titular context, A = Aesthetic context, S = Semantic context)

| ***Condition*** | ***Artist*** | ***Title*** | ***Year*** | ***Contextual Information*** |
| --- | --- | --- | --- | --- |
| A-T | Hilma af Klint | Altarpiece No. 1, Group X | 1915 |  |
| A-T | John Piper | Abstract I | 1935 |  |
| A-T | Joan Mitchell | South | 1989 |  |
| A-T | Max Ernst | Composition | 1914 |  |
| A-T | Wassily Kandinsky | Colour Study Squares with concentric circles | 1913 |  |
| F-T | Mary Adshead | The Cruise | 1934 |  |
| F-T | Vincent van Gogh | Self-Portrait | 1889 |  |
| F-T | Frida Kahlo | Self-Portrait with Thorn Necklace and Hummingbird | 1940 |  |
| F-T | Portrait by unknown artist | The Chandos | (1600-1610) |  |
| F-T | Sawlaram Haldankar | Glow of Hope | (1945-1946) |  |
| A-A | Jackson Pollock | Convergence | 1852 | Over the short 44 years of his life, Jackson Pollock painted an impressive 363 paintings. He is most famously known for his drip techniques, and many of these dynamic paintings that he produced have made their mark on the art world and can be claimed as notable. |
| A-A | Robert Motherwell | Elegy to the Spanish Republic | 1965 | Like other Abstract Expressionists, Motherwell was attracted to the Surrealist principle of automatism—of methods that escaped the artist's conscious intention—and his brushwork has an emotional charge, but within an overall structure of a certain severity. In fact Motherwell saw careful arrangements of color and form as the heart of abstract art, which, he said, is stripped bare of other things in order to intensify it, its rhythms, spatial intervals, and color structure."" |
| A-A | Piet Mondrian | Composition II in Red, Blue, and Yellow | 1930 | The initial composition was painted in Paris in 1937–38 and developed in New York between 1940 and 1942 when Mondrian added the red, blue and yellow colour fields. Mondrian also repositioned some of the black lines and added others. The horizontal lines were generally painted before the vertical lines. Mondrian is known to have used brushes, palette knives and a steel bar to achieve his sharp lines. |
| A-A | Gerhard Richter | Abstract painting | 1990 | The work initially consisted of four approximately even vertical bands – three dominated by differing shades of red, and one painted in a sharp green, although there were also blue and yellow streaks present. Once this composition had dried, the artist added thick daubs of additional paint to the surface, which he pressed, squeezed, and scraped across the pictorial plane in thick horizontal swipes using a long wooden ruler edged with rubber. |
| A-A | Henri Matisse | The Snail | 1953 | After 1948 Matisse was prevented from painting by ill health but, although confined to bed, he produced a number of works known as gouaches découpées. These were made by cutting or tearing shapes from paper which had been painted with gouache. The shapes were placed and pasted down by an assistant working under Matisse's instruction. Some of the later ones, such as The Snail, were of very large dimensions. |
| F-A | Edgar Degas | Dance Class at the Opera | 1872 | While Degas is known as an impressionistic artist, the piece holds many realist details to it. Degas preferred to refer to himself as a realist, even though his impressionist style greatly matched that of other French artists Pierre-Auguste Renoir and Claude Monet. Nevertheless, the piece intertwines both styles as exquisite detail is matched with delicate brush strokes. |
| F-A | George Seurat | A Sunday Afternoon on the Island of La Grande Jatte | (1884-1886) | This piece was created using the technique of Pointillism in which small, discrete dots work together to create a cohesive composition. Although this aesthetic approach was largely inspired by the dappled brushstrokes of Impressionism, the genre is in fact a branch of Post-Impressionism, a major movement that emerged in the 1890s. |
| F-A | Lauren Brevner | Iris | 2015 | Employing an eclectic range of mediums, her work blurs the line between cutting-edge contemporary painting and traditional Japanese arts and crafts. Opting for wooden panels instead of treated canvas, Brevner looks to the past when creating her paintings. Her ethereal portraits creatively combine oil and acrylic paint with glossy resin and glimmering silver and gold leaf. |
| F-A | Édouard Manet | Chez le père Lathuille (At the Père Lathuille Restaurant) | 1879 | Although ridiculed in his day for inadequate linear perspective, lack of spirituality, and controversial subject matter, Édouard Manet is considered by many art historians to be the father of Modernism. Technically his paintings are remarkable for their loose brushstrokes, nuanced color, unusual cropping, and sense of light. |
| F-A | Gustave Courbet | Le Désespéré (The Desperate Man) | (1843-1845) | The colours used in this painting point to Romanticism with the dark moody chromatic grey of the background, the black tie around his neck, his dark hair. Unlike the Romantics though, Courbet did not use smooth lines and soft forms. Instead, he used spontaneous brush strokes and created a roughness of paint texture. |
| A-S | Gheorghe Virtosu | Master of the waves | 2017 | The marine theme can be traced in many other details of the abstract painting, like the contrast of rectangular and curvy forms resembling the contrast between the permanence of ground and permanent changeability of water. \Moreover, in some of the cultures square is the symbol of the Earth element. Water is represented with the greyish-blue mass of the background. |
| A-S | Pablo Picasso | Fruit dish, bottle and violin | 1914 | Although the picture may seem to be entirely abstract, there are several recognisable objects. These include a table, an off-white tablecloth with grey tassels, the strings and neck of a violin, part of a newspaper (including the letters ‘AL’ of ‘JOURNAL’) and a dish of fruit. Picasso shows these objects from more than one point of view – for example, we see the table from the side and also look down at it from above. |
| A-S | Cy Twombly | Primavera | (1993-1995) | Primavera, or spring, represents the first season of the year. A column of red curved and slashed forms dominates the image. These relate to traditional Egyptian rowing boats which, it has been suggested, symbolise the journey through the underworld in the Egyptian ‘Book of the Dead’ (Bastian, p.37, note 15). Twombly lived for several months in Egypt in the mid 1980s and began to use the symbol of the boat in 1992. In Primavera, the red boat forms are smeared with patches of yellow, as though touched by the sun. |
| A-S | Joan Miró | Harlequin's Carnival | (1924-1925) | It depicts a festive, crowded scene where abstract characters seem to be caught up in a celebration. The figure depicted in the central-left portion of the canvas—with a half red, half-blue mask and diamond pattern on their tunic—references Italy’s commedia dell’arte. In this form of theater, the harlequin is a foolish character who is constantly unsuccessful in love. The hole in the character’s stomach perhaps references Miró’s own hunger and poverty at the time he created the painting. |
| A-S | Wyndham Lewis | Workshop | (1914-1915) | Here, Lewis uses angles and diagonals to suggest the geometry of modern buildings.Its harsh colours and lines echo the discordant vitality of the modern city in an ‘attack on traditional harmony’. |
| F-S | Emma Wesley | Johnson Gideon Beharry | 2006 | Lance Corporal Johnson Beharry  is a 27-year-old native of Grenada, who came to Britain in 1999 and joined the British Army in 2001.  He is the first living recipient since 1965 of the Victoria Cross for carrying out two individual acts of great heroism by which he saved the lives of his comrades. Both were in direct face of the enemy, under intense fire, at great personal risk to himself. |
| F-S | Chris Ofili | No Woman, No Cry | 1998 | The title of this work is the name of a 1974 song by the Jamaican reggae musician Bob Marley that entreats a female listener not to be sad. The phosphorescent inscription in the painting indicates that the crying woman depicted is Doreen Lawrence (now Baroness Lawrence of Clarendon OBE), the mother of Stephen Lawrence, who was murdered as a teenage boy in an unprovoked racist attack in London in 1993, and the photographs inside the tears in this work are all images of Stephen |
| F-S | Leonardo da Vinci | Lady with an Ermine | (1489-1491) | Many art historians identify the youthful woman in Lady with an Ermine as Cecilia Gallerani, the mistress of Leonardo’s patron, Ludovico Sforza, duke of Milan. The ermine was often used as an emblem for the duke. The woman turns her head to the right, her bright eyes seemingly directed toward something outside the frame. Although the painting has been heavily overpainted, notably the dark background, it nonetheless reveals Leonardo’s knowledge of anatomy and his ability to represent character in posture and expression. |
| F-S | Botticelli | Primavera | (1470-1980) | Although the complex meaning of the composition remains a mystery, the painting is a celebration of love, peace, and prosperity. This painting, usually known as the Primavera [or ‘Spring’] shows nine figures from classic mythology advancing over a flowery lawn in a grove of orange and laurel trees. In the foreground, to the right, Zephyrus embraces a nymph named Chloris before taking her; she is then portrayed after her transformation into Flora, the spring goddess. The centre of the painting is dominated by the goddess of love and beauty, Venus, chastely dressed and set slightly back from the others, and by a blindfolded Cupid, firing his arrow of love. |
| F-S | M. C. Escher | Hand with reflecting sphere | 1935 | The work is representative of the artist’s increasing fascination with visual illusions, mirrored reflections, and perceptual self-references. The plain background of the work focuses attention onto the reflection but also causes the viewer to question the accuracy of the depiction, the hand and sphere appear to exist in a void in which only the reflection is real. This enigma is further enhanced by the fact that Escher gazes directly out of the picture instead of representing himself drawing the image. The fact that his face appears directly in the centre of the sphere indicates his mastery over the illusion. |

***Online Pilot Study***

We ran an online pilot study hosted by Qualtrics using the same material as described in the main paper. We collected the data of 134 participants recruited either through the Durham University Psychology Department Participant Pool, whereby participants received credit for their participation, or through personal contact. Participant ages ranged from 18 to 87yrs (*M*= 26.42, *SD*= 13.66), 102 identified as female.

All measures were analysed using a 2 (genre) * 3 (contextual information) repeated measures ANOVA. For post-hoc analysis we used the Bonferroni method for multiple comparisons and cases where Mauchly’s test of sphericity indicated a violation of the assumption of sphericity we used the Greenhouse-Geisser correction. All descriptive and inferential statistics were performed using JASP 0.16.3 (2022).

*Liking/interest results*

We found a significant main effect of context (*F*(1.79, 237.42) =7.93, *p*< .001, η^2^_p_ = .56), a significant main effect of artistic genre (*F*(1, 133) = 156.40, *p*<.001, η^2^_p_ = .54) and a significant context * artistic genre interaction effect (*F*(1.80, 239.49) = 80.08, *p* <.001, η^2^_p_ = .38) (see Figure 1).

The significant interaction was analysed using two one-way ANOVAs. Comparisons of figurative artworks showed a significant difference between contexts (*F*(2,266)=33.82, *p*< 001, η^2^ = .20). Post hoc paired samples t-tests showed that titular artworks were rated as significantly less liked/interesting compared to artworks in both aesthetic (*t*(133)=8.28,*p* < 001), and semantic contexts (*t*(133)= 4.86, *p* <.001). Liking/ interest ratings for artworks in the aesthetic context were significantly higher than for those in the semantic context (*t*(133)= 3.48, *p*=.001).

Comparisons of abstract artworks showed a significant difference between contexts, (*F*(1.73,229.55) =53.13, *p*< 001, η^2^_p_ = .29). Post hoc paired samples t-tests showed that titular artworks were rated as significantly more liked/ interesting compared to artworks in both aesthetic(*t(*133)= 9.88, *p*< 001), and semantic contexts, (*t*(133)=247, *p*= 015). Liking/ interest ratings for artworks in the aesthetic context were however significantly lower than for those in the semantic context (*t*(133)= 6.63,*p* < 001).

**Figure** **1**

*Average liking/interest scores for titular, aesthetic, and semantic contextual information separately for figurative and abstract artworks.*


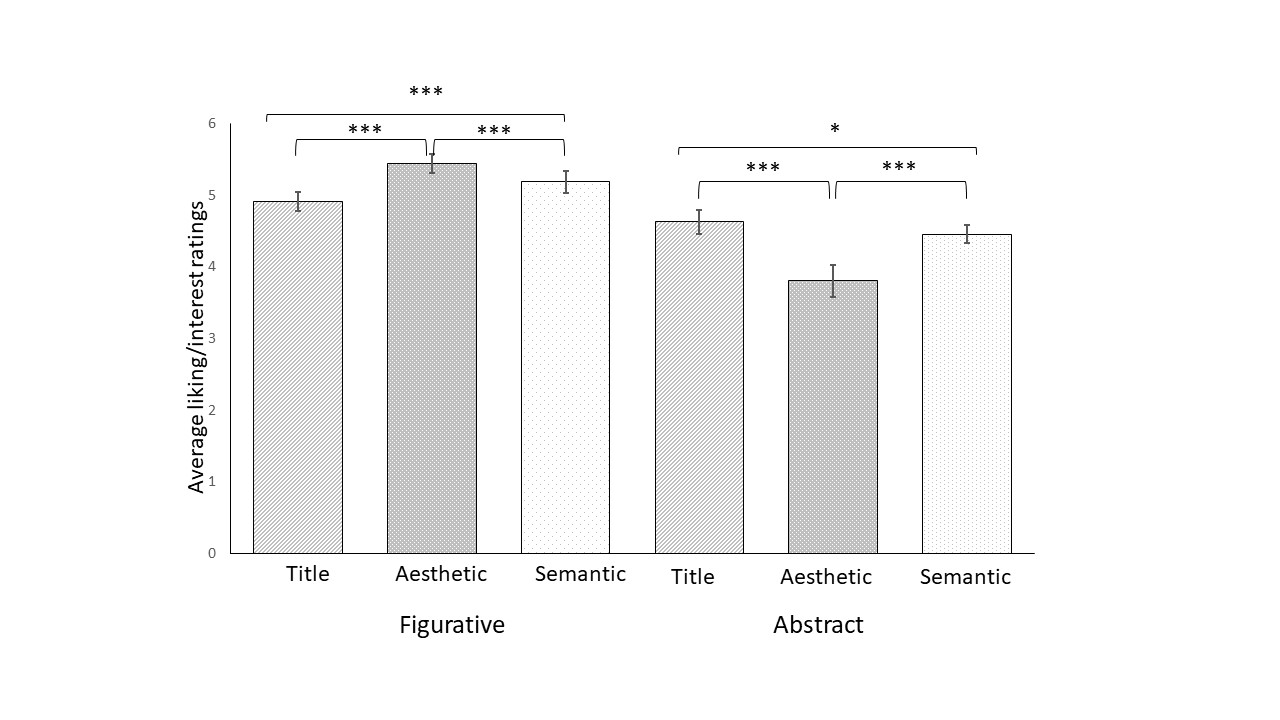


*Note: Error bars show 95% confidence intervals - *: p<.05, **: p<.01, ***: p<.001*

*Understanding results*

Results of the 2*3 ANOVA revealed a significant main effect of context (*F*(1.91, 253.66) = 79.36, *p*< .001, η^2^_p_ = .37) as well as a significant main effect of artistic genre (*F*(1, 133) = 564.78, *p*< .001, η^2^_p_ = .81) on understanding ratings. Similarly to liking/interests, there was a significant context * artistic genre interaction effect (*F*(2, 266) = 105.42, *p* < 001, η^2^_p_= .44). Results are summarized on Figure 2.

Comparisons of figurative artworks showed a significant effect of context on understanding (*F*(2, 266) =32.61, *p*< 001, η^2^ = .20). Post hoc paired samples t-tests using the Bonferroni correction showed that titular artworks were significantly less understood compared to artworks in both aesthetic (*t*133)=8.34,*.p* < 001), and semantic contexts, (*t(*133)= 5.46, *p* < 001). However, understanding ratings for artworks in the aesthetic context were not significantly different than for those in the semantic context (*t*(133)= 1.66, *p* = .100).

Comparisons of abstract artworks showed there were significant difference between contexts (*F*(2,266) =120.56, p< 001, η^2^ = .48). Post hoc paired samples t-tests showed that titular artworks were rated as significantly more understood compared to artworks in the aesthetic context (*t*(133)= 4.04, *p*< .001), but less understood than artworks in semantic contexts, (*t*(133)= 11.17, *p*< 001). Understanding ratings for artworks in the aesthetic context were also significantly lower than for those in the semantic context (*t*(133)= 14.92, *p* < 001).

**Figure 2**

*Average understanding scores for titular, aesthetic, and semantic contextual information separately for figurative and abstract artworks*


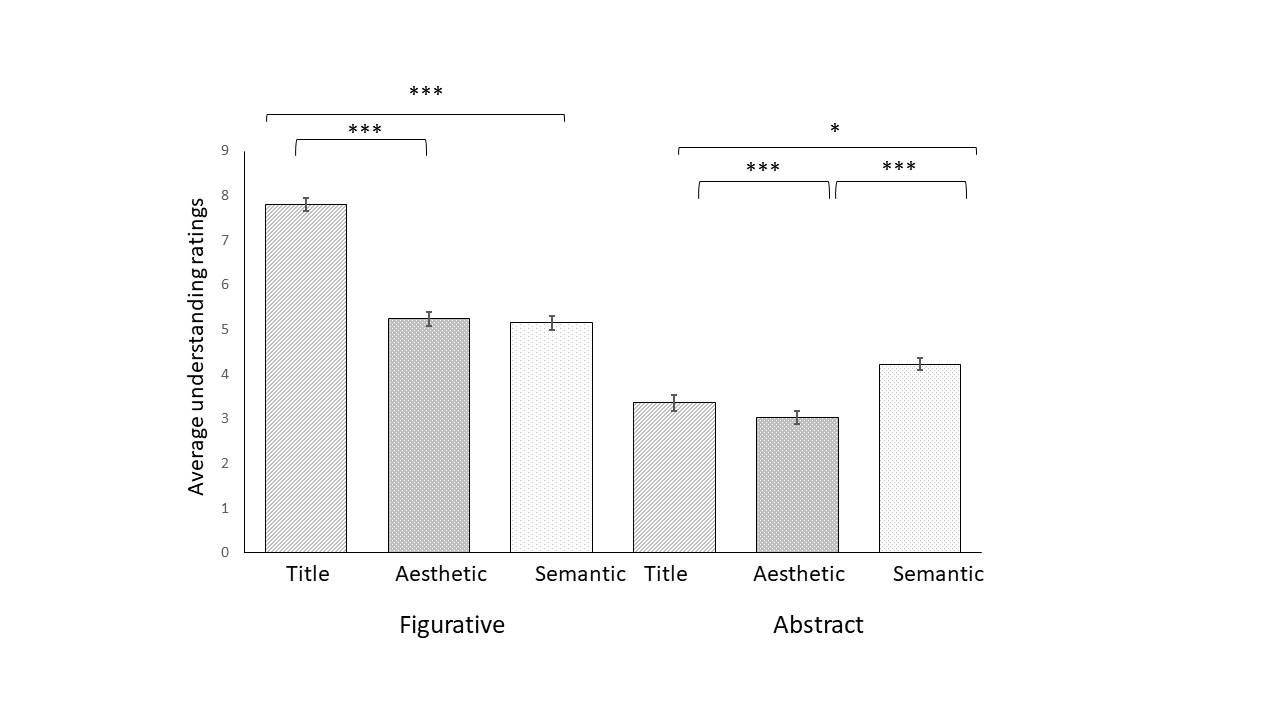


*Note: Error bars show 95% confidence intervals - *: p<.05, **: p<.01, ***: p<.001*

**Table 2**

*Median familiarity ratings and standard deviation for each artwork*

| ***Artwork*** | ***Average Familiarity Rating*** | ***Standard Deviation*** |
| --- | --- | --- |
| Altarpiece No. 1 | 1 | 1.00 |
| Abstract I | 1 | 0.99 |
| South | 1 | 0.60 |
| Composition | 1 | 0.58 |
| Colour Study Squares with concentric circles | 1.5 | 1.81 |
| The Cruise | 1 | 0.72 |
| Self-Portrait* | 5 | 0.58 |
| Self-Portrait with Thorn Necklace and Hummingbird | 2.5 | 1.77 |
| The Chandos | 4.5 | 1.38 |
| Glow of Hope | 1 | 0.51 |
| Master of the waves | 1 | 0.53 |
| Fruit dish | 2 | 1.37 |
| Primavera | 1 | 0.68 |
| Harlequin's Carnival | 1 | 1.39 |
| Workshop | 1 | 0.93 |
| Johnson Gideon Beharry | 1 | 0.38 |
| No Woman | 1 | 1.01 |
| Lady with an Ermine | 2 | 1.51 |
| Primavera | 2 | 1.42 |
| Hand with reflecting sphere | 1.5 | 1.63 |
| Convergence | 2 | 1.45 |
| Elegy to the Spanish Republic | 1 | 0.38 |
| Composition II in Red* | 5 | 1.38 |
| Abstract painting | 1 | 0.84 |
| The Snail | 2.5 | 1.78 |
| Dance Class at the Opera | 1.5 | 1.39 |
| A Sunday Afternoon on the Island of La Grande Jatte* | 5 | 1.55 |
| Iris | 1 | 0.51 |
| Chez le père Lathuille (At the Père Lathuille Restaurant) | 1 | 1.07 |
| Le Désespéré (The Desperate Man) | 1 | 1.49 |

*artwork that had a median familiarity ratings of 5

***Statistical analysis without highly familiar paintings***

We ran the main analysis (average liking/interest, understanding and total exploration time) without the artworks that received a median familiarity rating of 5.

*Liking/Interest results*

Descriptives statistics (mean and standard deviation) are summarized in Table 3

Results of the 2*3 repeated measures ANOVA revealed a significant main effect of artistic genre (*F*(1, 29) = 21.33, *p*<.001, η^2^_p_ = .42) and a significant context * artistic genre interaction effect (*F*(2,58) = 10.64, *p* <.001, η^2^_p_ = .27), the main effect of context was not significant (*F*(2,58) = 2.84, *p* =.067, η^2^_p_ = .27).

**Table 3**

*Descriptive statistics for the liking/interest ratings*

|  | | | | | | | | | | | | | |
| --- | --- | --- | --- | --- | --- | --- | --- | --- | --- | --- | --- | --- | --- |
|  | | **Abstract** | | | | | | **Figurative** | | | | | |
|  | | **Titular** | | **Aesthetic** | | **Semantic** | | **Titular** | | **Aesthetic** | | **Semantic** | |
| Mean |  | 4.34 |  | 4.15 |  | 4.48 |  | 4.70 |  | 5.31 |  | 4.99 |  |
| Std. Deviation |  | 0.84 |  | 1.02 |  | 1.01 |  | 0.86 |  | 0.83 |  | 0.67 |  |
|  | | | | | | | | | | | | | |

*Understanding*

Descriptives statistics (mean and standard deviation) are summarized in Table 4.

Results of the 2*3 repeated measures ANOVA revealed a significant main effect of artistic genre (*F*(1, 29) = 88.28, *p*<.001, η^2^_p_ = .75) and a significant main effect of context (*F*(2,58) = 24.61, *p*<.001, η^2^_p_ = .46). The interaction context * artistic genre did not reach significance (*F*(2,58) = 2.97, *p* =.059, η^2^_p_ = .09).

**Table 4**

*Descriptive statistics for the understanding ratings*

|  | | | | | | | | | | | | | |
| --- | --- | --- | --- | --- | --- | --- | --- | --- | --- | --- | --- | --- | --- |
|  | | **Abstract** | | | | | | **Figurative** | | | | | |
|  | | **Titular** | | **Aesthetic** | | **Semantic** | | **Titular** | | **Aesthetic** | | **Semantic** | |
| Mean |  | 2.90 |  | 3.03 |  | 3.65 |  | 4.39 |  | 4.67 |  | 4.81 |  |
| Std. Deviation |  | 0.92 |  | 1.19 |  | 1.05 |  | 0.90 |  | 0.98 |  | 0.91 |  |
|  | | | | | | | | | | | | | |

*Exploration time*

For the median exploration time, the ANOVA analysis revealed a significant main effect of artistic genre (*F*(1, 29) = 5.78, *p*=.023, η^2^_p_ = .17, a significant main effect of context (*F*(1.67, 48.43) = 4.98, *p=*.015, η^2^_p_ = .18) as well as a significant context * artistic genre interaction (*F*(2,58) = 3.47, *p* =.038, η^2^_p_ = .11). Descriptives are summarized in Table 5.

**Table 5**

*Descriptive statistics for the exploration time (in seconds)*

|  | | | | | | | | | | | | | |
| --- | --- | --- | --- | --- | --- | --- | --- | --- | --- | --- | --- | --- | --- |
|  | | **Abstract** | | | | | | **Figurative** | | | | | |
|  | | **Titular** | | **Aesthetic** | | **Semantic** | | **Titular** | | **Aesthetic** | | **Semantic** | |
| Mean |  | 22.85 |  | 20.24 |  | 25.44 |  | 24.08 |  | 25.76 |  | 26.27 |  |
| Std. Deviation |  | 12.71 |  | 12.45 |  | 14.67 |  | 12.21 |  | 13.71 |  | 14.14 |  |
|  | | | | | | | | | | | | | |

**Figure 3**

*Examples of fixation maps for the final sample size (N = 30).*

***
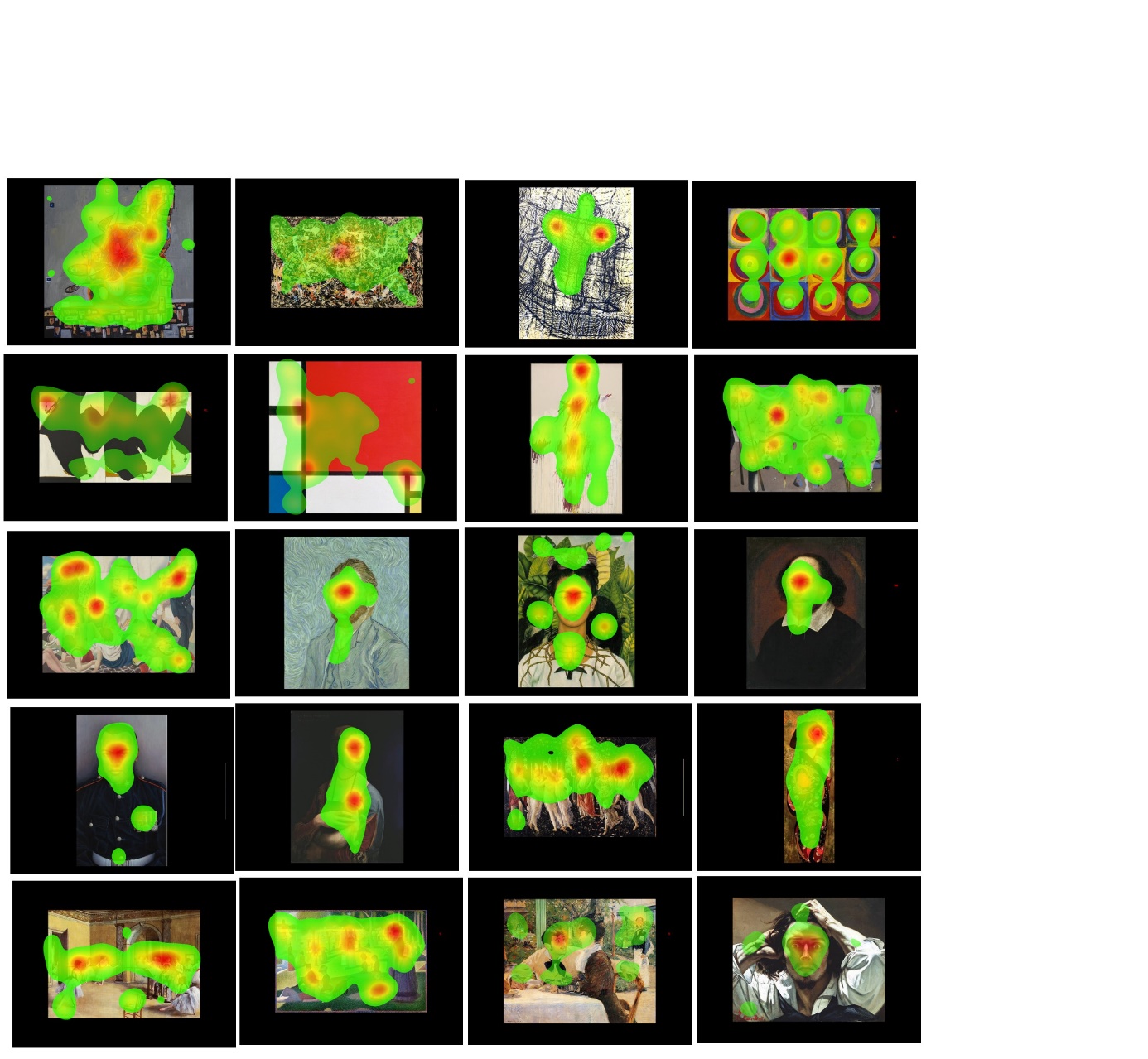
***

List of depicted artworks authors, titles, year, copyrights, and sources:

Gheorghe Virtosu Master of the waves 2017 (© V I RTO S U A RT, source: Virtosu Art Gallery)

Jackson Pollock Convergence 1952 (© The Pollock-Krasner Foundation ARS, NY and DACS, London 2024, source: Wikiart)

Max Ernst Composition (un chant d’amour) 1958 (© ADAGP, Paris and DACS, London 2024, source : Art Institute Chicago,

Wassily Kandinsky Colour Study Squares with concentric circles 1913 (public domain, source: Wikimedia Commons)

Robert Motherwell Elegy to the Spanish Republic 1965 (©The Solomon R. Guggenheim Foundation/Art Resource, NY/ Scala, Florence, Source: Scala group)

Piet Mondrian Composition II in Red, Blue, and Yellow 1930 (public domain, source: Wikimedia Commons)

Cy Twombly Primavera (1993-1995) (© Cy Twombly Foundation, source: Tate )

Joan Miró Harlequin's Carnival (1924-1925) (© Successió Miró / ADAGP, Paris and DACS London 2024, source: Wikiart)

Mary Adshead The Cruise 1934 (© Estate of Mary Adshead. All Rights Reserved, DACS 2024, source: Tate)

Vincent van Gogh Self-Portrait 1889 (public domain, source: Wikimedia Commons)

Frida Kahlo Self-Portrait with Thorn Necklace and Hummingbird 1940 (© Banco de México Diego Rivera Frida Kahlo Museums Trust, Mexico, D.F. / DACS 2024, source: Wikipedia)

Portrait by unknown artist The Chandos (1600-1610) (public domain, source: Wikimedia Commons)

Emma Wesley Johnson Gideon Beharry 2006 (© National Portrait Gallery, London, source: National Portrait Gallery)

Leonardo da Vinci Lady with an Ermine (1489-1491) (public domain, source: Wikimedia Commons)

Botticelli Primavera (1470-1980) (public domain, source: Wikimedia Commons)

Lauren Brevner Iris 2015 (© Lauren Brevner, source: Lauren Brevner)

Edgar Degas Dance Class at the Opera 1872 (public domain, source: Wikimedia Commons)

George Seurat A Sunday Afternoon on the Island of La Grande Jatte (1884-1886) (public domain, source: Wikimedia Commons)

Édouard Manet Chez le père Lathuille (At the Père Lathuille Restaurant) 1879 (public domain, source: Wikimedia Commons)

Gustave Courbet Le Désespéré (The Desperate Man) (1843-1845) (public domain, source: Wikimedia Commons)
